# Demenz mit Lewy-Körpern: alte und neue Erkenntnisse – Teil 2: Behandlung

**DOI:** 10.1007/s00115-023-01577-2

**Published:** 2023-12-14

**Authors:** Richard Dodel, Daniela Berg, Thomas Duning, Elke Kalbe, Philipp T. Meyer, Alfredo Ramirez, Alexander Storch, Dag Aarsland, Frank Jessen

**Affiliations:** 1 Lehrstuhl für Geriatrie, Universität Duisburg-Essen, Virchowstraße 171, 45147 Essen, Deutschland; 2 Neurologische Klinik, Universität Kiel, Kiel, Deutschland; 3 Neurologische Klinik, Universität Münster, Münster, Deutschland; 4 Medizinische Psychologie, Neuropsychologie und Gender Studies & Centrum für Neuropsychologische Diagnostik und Intervention (CeNDI), Universität Köln, Köln, Deutschland; 5 Klinik für Nuklearmedizin, Universitätsklinikum Freiburg, Medizinische Fakultät, Albert-Ludwigs-Universität Freiburg, Freiburg, Deutschland; 6 Klinik und Poliklinik für Psychiatrie und Psychotherapie, Universität Köln, Köln, Deutschland; 7 Klinik für Neurologie, Universität Rostock, Rostock, Deutschland; 8 Centre for Age-Related Medicine (SESAM), Stavanger University Hospital, Stavanger, Norway; Institute of Psychiatry, Psychology, and Neuroscience, King’s College London, London, Großbritannien

**Keywords:** Behandlung, Cholinesterase-Hemmer, Verhaltens- und psychische Symptome, Demenz, Parkinson-Krankheit mit Demenz, Treatment, Cholinesterase inhibitors, Behavioral and psychological symptoms, Dementia, Parkinson’s disease with dementia

## Abstract

**Hintergrund:**

Die Behandlung von Patienten mit Demenz mit Lewy-Körpern (DLK) ist vielschichtig, da motorische Symptome, kognitive Symptome und Verhaltens- und psychische Symptome in unterschiedlichen Konstellationen auftreten können. Zudem ist der Einsatz bestimmter Medikamente nur bedingt möglich (z. B. Neuroleptika).

**Ziel der Arbeit:**

In diesem Übersichtsartikel sollen die wichtigsten neuen Erkenntnisse zur Behandlung der DLK zusammengetragen werden.

**Ergebnisse:**

Bisher existiert in Deutschland keine zugelassene Therapieoption für die Behandlung der Patienten mit DLK; die Evidenzbasis für die pharmakologischen und nichtpharmakologischen Behandlungsoptionen ist zudem dürftig. Die derzeit konsentierten Therapieansätze stützen sich auf die Behandlung der motorischen Symptome in Anlehnung an die Therapie bei der Parkinson-Krankheit und bei den Verhaltenssymptomen an die Therapie der Alzheimer-Krankheit.

**Diskussion:**

Die Behandlung der DLK mit ihren verschiedenartigen Symptomen ist schwierig und oftmals erst in enger fachärztlicher Zusammenarbeit adäquat für den Patienten zu erreichen.

## Abstract (en)

**Background:**

The treatment of patients with dementia with Lewy bodies (DLB) is multifaceted, as motor symptoms, cognitive symptoms, behavioral and psychological symptoms can occur in different constellations. In addition, the use of certain medications is limited (e.g., neuroleptics).

**Objective:**

To summarize the main recent findings on the treatment of DLB.

**Results:**

To date, there is no approved therapeutic option for the treatment of patients with DLB in Germany; moreover, the evidence base for pharmacological and non-pharmacological treatment is sparse. The currently consented treatment options are based on the treatment of motor symptoms in the same way as the treatment of Parkinson’s disease and for behavioral symptoms based on the treatment for Alzheimer’s disease.

**Discussion:**

The treatment of DLB with its various symptoms is difficult and often can only be adequately achieved for the patient in close cooperation with a specialist.

Die Behandlung der Demenz mit Lewy-Körpern (DLK) orientiert sich an den Zielsymptomen. Man unterscheidet nach (1) den motorischen Beschwerden, (2) der kognitiven Leistungseinbuße und (3) den psychischen Störungen und Verhaltensauffälligkeiten. Die adäquate Behandlung stellt insbesondere in fortgeschrittenen Stadien der Erkrankung eine Herausforderung dar, die oft nur im fachärztlichen Kontext hinreichend gemeistert werden kann. Bisher stehen allerdings nur vereinzelt kontrollierte klinische Studien zur Behandlung von Patienten mit DLK zur Verfügung, ein evidenzbasiertes Vorgehen ist daher zum gegenwärtigen Zeitpunkt nur sehr bedingt möglich. Erschwerend kommt hinzu, dass in den klinischen Studien Patienten mit Parkinson-Krankheit mit Demenz (PKD) und DLK oft nicht unterschieden wurden. Wo dies getan wurde, kamen die Studien sogar zu unterschiedlichen Ergebnissen.

Insgesamt führen diese Schwierigkeiten nach heutigem Kenntnisstand zu einer Unterversorgung von Patienten mit DLK mit spezifischen Therapien. So erhielten in einer Erhebung in Pflegheimen in Schweden lediglich ca. 45 % der DLK-Patienten mit Demenz eine Therapie mit einem Antidementivum [28]. In derselben Studie erhielten 22 % der Patienten ein Antipsychotikum und 50 % ein Antidepressivum, aber 60 % ein Anxiolytikum (Benzodiazepine) und sogar 41 % ein Sedativum. Auch wenn hier keine direkte Korrelation der Medikation mit den Symptomen erfolgte (wie bei der Demenz), ist doch eine Unter- oder Fehlversorgung mit Pharmakotherapie bei diesen Patienten anzunehmen.

## Behandlung der motorischen Symptome

Derzeit liegen keine kontrollierten klinischen Studien vor, die eine Behandlung der motorischen Symptome bei der DLK systematisch untersucht haben. Ersatzweise gelten die Regeln wie bei der Behandlung multimorbider Patienten mit Parkinson-Krankheit [13]. Allerdings sprechen Patienten mit DLK häufig schlechter auf L‑Dopa als Patienten mit Parkinson-Krankheit an. Eine Verbesserung durch die Gabe von Levodopa (d. h. eine mindestens 10 %ige Verbesserung der UPDRS-III) konnte nur bei 32–50 % der DLK-Patienten im Vergleich zu 65–70 % bei Patienten mit PKD beobachtet werden [3].

Ein Behandlungsbeginn ist dann indiziert, wenn die motorischen Symptome mit den Aktivitäten des täglichen Lebens des individuellen Patienten interferieren. Es sollte dann mit einer niedrigen Dosis (z. B. 50 mg L-Dopa/Dopadecarboxylaseinhibitor) begonnen werden und nur sehr langsam gesteigert werden, da vermehrt psychotische Symptome auftreten können [10]. Ein Therapieversuch mit bis zu 600–800 mg (maximal 1000 mg) L‑Dopa sollte unternommen werden, sofern der Patient dies toleriert, da sich bei der DLK ein Ansprechen gelegentlich erst in hohen Dosisbereichen einstellen kann.

Der Einsatz von Dopaminagonisten sollte nicht oder nur sehr vorsichtig erfolgen, da bei Dopaminagonisten oft bereits in niedrigen Dosen eine Zunahme oder das Neuauftreten von Halluzinationen und auch eine vermehrte Tagesmüdigkeit beobachtet werden. Die Autoren vermeiden daher diese Substanzgruppe, wenn möglich.

Der Zusatz von COMT-Hemmern zu einer Levodopabehandlung kann hingegen zwecks Verstärkung und Verlängerung der L‑Dopa-Wirkung sinnvoll sein und ist in der Regel sicher. In aller Regel wird hier Entacapon gewählt, zum recht neu verfügbaren Opicapon liegen noch keine Erfahrungen vor (Opicapon kann eine Alternative sein, falls unter Entacapon eine Diarrhö auftritt). Eine Anpassung bzw. Reduktion der L‑Dopa-Tagesdosis kann erforderlich sein. Auf die Entwicklung psychotischer Symptome ist engmaschig zu achten.

MAO-B-Hemmer werden wegen der Gefahr der Entwicklung psychotischer Symptome und der möglichen Interaktion mit Antidepressiva in dieser Indikation nicht empfohlen. Das Antiepileptikum Zonisamid als Adjunkt zu L‑Dopa verbessert dosisabhängig die Motorik bei Patienten mit DLK und zeigt eine gute Verträglichkeit [25]. Der Wirkmechanismus ist ungeklärt. In Japan ist Zonisamid in der Indikation DLK zugelassen, nicht jedoch in der EU/Deutschland.

## Behandlung der kognitiven Leistungseinbuße

Der Einsatz von Cholinesteraseinhibitoren (CHEI) erklärt sich durch den Verlust cholinerger Neurone in dem Nucleus basalis von Meynert und eine niedrige Cholinacetyltransferaseaktivität [23]. Darüber hinaus sind aber mehr postsynaptische Muskarin- und Nikotinrezeptoren im Hirnstamm und dem basalen Vorderhirn erhalten.

Bisher ist in Deutschland kein Medikament für die Behandlung der DLK zugelassen worden. In Japan und den Philippinen ist Donepezil bei DLK zugelassen. Rivastigmin ist für den Einsatz bei der PKD in Europa, Kanada und den USA lizenziert. Derzeit stehen in Deutschland als Cholinesteraseinhibitoren Donepezil, Rivastigmin und Galantamin zur Verfügung. Die Daten zur Evidenz für den Einsatz dieser Substanzen ist in mehreren Übersichtsartikeln zusammengefasst [12, 18]. Im Einzelnen sollen nur die neueren Studien kurz besprochen werden.

### Donepezil

Zum gegenwärtigen Zeitpunkt steht die beste Evidenz für den Einsatz von Donepezil bei der DLK zur Verfügung. In einer 12-wöchigen placebokontrollierten Phase-II-Studie war Donepezil in der Dosierung von 5 und 10 mg/Tag signifikant besser als Placebo hinsichtlich des MMSE, dem CIBIC-plus und hinsichtlich der Verhaltenssymptomatik (NPI; [20]). An Nebenwirkungen traten auf: gastrointestinale Nebenwirkungen unter der Therapie mit 5 mg (30,3 %) und 10 mg (35,1 %; Placebo: 23,5 %), Exazerbation der Parkinson-Symptomatik unter der Therapie mit 5 mg (12,1 %) und 10 mg (2,1 %; Placebo: 2,9 %), psychiatrische Symptome unter der Therapie bei 5 mg (11,8 %) und 10 mg (5,2 %; Placebo: 8,1 %). In der Open-label-Extension der Studie über einen Zeitraum von 52 Wochen unter einer Dosis von 5 mg fanden sich verbesserte Werte über den gesamten Behandlungszeitraum für die Outcomes MMSE und NPI. Die Langzeittherapie führte zu keinen weiteren Nebenwirkungen.

In einer weiteren placebokontrollierten Phase-III-Studie über 12 Wochen derselben Arbeitsgruppe konnten die Ergebnisse für den MMSE bei einer Dosierung von 10 mg, nicht aber in der niedrigeren Dosierung von 5 mg reproduziert werden; für die Zielgröße „Verhaltensstörungen“ konnte keine Signifikanz nachgewiesen werde [14]. Die Nebenwirkungsrate für das Auftreten von Parkinson-Symptomen wurde bei 10 mg mit 8,2 % angegeben (5 mg: 4,3 %; Placebo: 4,3 %). In der Open-label-Extension dieser Studie unter einer Dosis von 10 mg Donepezil konnte eine kognitive Funktionsverbesserung für den Zeitraum von 52 Wochen aufrechterhalten werden [21]. Die Auswertung der vorgenannten Studien erbrachte, dass unter Donepezil keine schwerwiegende Exazerbation von Parkinson-Symptomen beobachtet wurde; vorsichtiges und langsames Einschleichen wird jedoch bei empfohlen [21].

### Rivastigmin

Für das Medikament Rivastigmin liegen keine neuen placebokontrollierten Studien vor. Das Medikament kann für die Behandlung der DLK im Sinne eines Heilversuches eingesetzt werden. Für die Hochdosispflastertherapie mit 13,3 mg/24 h, die für die Behandlung der AD eingesetzt wird, liegen bisher für die Indikation DLK keine klinischen Daten vor [9]. Die Substanz ist für die Indikation PKD geprüft und zugelassen. Das gilt nicht für die Pflasterformulierung, die Verschreibung wird aber von den Kassen meist erstattet.

### Galantamin

In einer kleinen Open-label-Studie mit 50 DLK-Patienten bis 24-mg-Tagesdosis Galantamin konnte nach 24 Wochen ein signifikanter Effekt im CGIC sowie eine Reduktion im NPI-12 nachgewiesen werden [8]. Weitere Studien liegen gegenwärtig nicht vor [15]. Galantamin ist in Deutschland für die Behandlung der DLK und PKD nicht zugelassen. Es handelt sich um eine Off-label-Behandlung.

### Memantine

Zwei ältere Studien liegen für den Einsatz von Memantine bei DLK vor [16]. Memantine wurde in beiden Studien gut vertragen. Neuere Auswertungen der Studien im Hinblick auf episodisches Gedächtnis und Aufmerksamkeit zeigten für die Dosis von 20 mg/Tag einen signifikanten Effekt [27]. In einer 36-Monate-Follow-up-Studie konnte bei Patienten mit positivem Effekt auf Memantine eine verlängerte Überlebenszeit beobachtet werden. Aufgrund der geringen Zahlen ist aber keine valide Aussage möglich [24]. Eine Zulassung, wie für die AD, besteht in Deutschland gegenwärtig nicht; es handelt sich um eine Off-label-Behandlung.

## Behandlung der psychischen und Verhaltens-symptome

Psychische Störungen und Verhaltenssymptome werden bei Patienten mit AD in vier Symptomcluster differenziert: (1) affektive Symptome (Depression, Angst), (2) Hyperaktivität (Agitation, Euphorie, Enthemmung, Irritierbarkeit, auffälliges motorisches Verhalten), (3) psychotische Symptome (Wahn, Halluzination) und (4) Apathie (Apathie, Essstörungen). Diese Einteilung wird hier für die DLK übernommen.

### Psychosoziale Interventionen und nichtpharmakologische Behandlungsstrategien

Psychosoziale Interventionen und nichtpharmakologische Behandlungsstrategien sind zentraler und notwendiger Bestandteil in der Behandlung von psychischen und Verhaltenssymptomen bei Demenz. Ansätze und Ziele dieser Verfahren sind breiter als die pharmakologischer Therapien und zielen zusätzlich auch auf die Prävention der genannten Symptome ab. Entsprechend den Rahmenempfehlungen zum Umgang mit herausforderndem Verhalten (BMG, 2006) werden folgende Verfahren vorgeschlagen:

- verstehende Diagnostik zur Identifizierung von Bedingungsfaktoren,
- Einsatz von Assessment-Instrumenten zur systematischen Aufdeckung und Dokumentation herausfordernden Verhaltens,
- validierendes Verhalten,
- Erinnerungspflege,
- basale Stimulation, Snoezelen, körperliche Berührung,
- Bewegungsförderung,
- Handeln in Krisensituationen.

Für die DLK liegen darüber hinaus zu nichtpharmakologischen Interventionen nur vereinzelt Studien vor. In kürzlich publizierten Übersichtsarbeiten wurden die Ergebnisse zusammengetragen, allerdings wird keine scharfe Trennung zwischen Patienten mit PKD oder DLK getroffen [11, 22]: Studien mit physiotherapeutischer Beübung wiesen auf Verbesserungen bei motorischen Defiziten hin. Kognitive Rehabilitation führte zu signifikanten Verbesserungen der Stimmung, der Kognition, der gesundheitsbezogenen Lebensqualität und Zufriedenheit der Patienten. Unter Lichttherapie wurde ein partieller Trend bezüglich der Verbesserungen der Stimmung und der Schlafqualität beschrieben.

### Affektive Symptome

Es liegen keine randomisierten klinischen Studien zur pharmakologischen Behandlung affektiver Symptome bei Patienten mit DLK vor. Grundsätzlich sollen Antidepressiva mit anticholinerger Komponente, wie z. B. trizyklische Antidepressiva, vermieden werden. Zu bevorzugen sind Serotoninwiederaufnahmehemmer, duale Antidepressiva (serotonerg/noradrenerg oder dopaminerg/noradrenerg) oder das noradrenerg und spezifisch serotonerg wirkende Antidepressivum Mirtazapin. Die Auswahl des Medikaments orientiert sich dabei an der Zielsymptomatik, möglicher Interaktionen mit der bestehenden Parkinson-Medikation (cave: MAO-B-Hemmer), der Zusatzmedikation (z. B. Marcumar), den verschiedenen vorliegenden Begleiterkrankungen sowie der gewünschten Zusatzwirkung, z. B. Schlafinduktion. Eine engmaschige Kontrolle möglicher Nebenwirkungen z. B. in Bezug auf die kardiale Erregungsleitung oder den Elektrolytstoffwechsel ist erforderlich.

#### Angst

Angstsymptome, wie innere Anspannung, Befürchtungen und Nervosität sind häufig bei Patienten mit DLK zu beobachten [4]. Häufig treten sie mit Symptomen der Depression auf. Es existieren derzeit keine größeren randomisierten Studien, die Angstsymptome als primären Endpunkt untersucht haben. Antidepressiva werden auch zur Therapie von Angststörungen eingesetzt und eine Behandlung von Angstsymptomen bei Patienten DLK ist vertretbar. Benzodiazepine sollten vermeiden werden.

### Apathie

Antriebsstörungen und Verlust von Initiative können auch ohne gedrückte Stimmung auftreten und werden dann eigenständig mit dem Begriff der Apathie bezeichnet. Die Apathie verschlechtert den Krankheitsverlauf, verhindert die Teilnahme von Patienten am Alltagsleben und führt zu einer Belastung der Pflegenden. Bei DLK-Patienten tritt Apathie mit einer Häufigkeit von ca. 60 % auf [4]. Es liegen keine Therapiestudien zur Apathie bei Patienten mit DLK vor. Im Bereich der AD gibt es randomisierte klinische Studien zur Behandlung der Apathie mit Methylphenhydat, die eine Wirksamkeit zeigen [19]. Eine direkte Behandlungsempfehlung für die DLK leitet sich hieraus aber nicht ab.

### Psychotische Symptome

**Figure 1:**
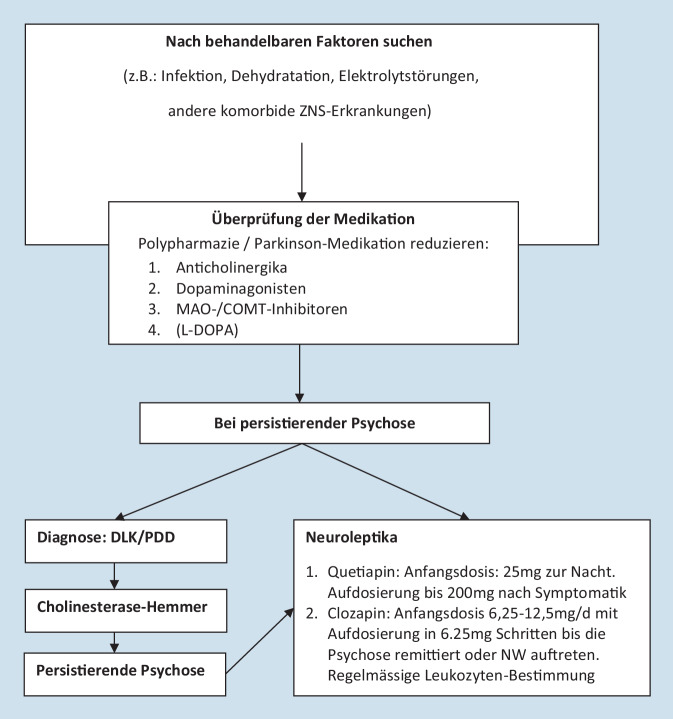

Halluzinationen und Wahn finden sich bei bis zu 75 % der an DLK erkrankten Patienten auch schon sehr früh im Krankheitsverlauf und damit deutlich häufiger als bei der AD oder der PK und mit Demenz [1]. Als Risikofaktoren für eine Psychose gelten: Parkinson-Medikamente, hohes Alter, Polypharmazie sowie andere psychische und Verhaltenssymptome wie Depression, Angst und Schlafstörungen [26]. Es wird empfohlen eine mögliche Medikation zur Behandlung von Parkinson-Symptomen zunächst anzupassen (Abb. 1).

Zur pharmakologischen Behandlung von Halluzinationen bei DLK gibt es Hinweise auf eine klinisch relevante Wirksamkeit für Cholinesterasehemmer, insbesondere für Rivastigmin. Da diese Medikation auch für die Behandlung der kognitiven Beeinträchtigung (off-label) einsetzt werden kann, kommt es hierbei häufig auch zu einer Verbesserung von Halluzinationen.

Bei Patienten mit DLK ist die Gabe von Neuroleptika sehr problematisch, da schwere Reaktionen unter einer solchen Behandlung auftreten können. Nebenwirkungen unter Neuroleptika wurden sogar als zusätzliches „unterstützendes Kriterium“ der Diagnose der DLK vorgeschlagen. Neuroleptisch wirksame Substanzen können bei der DLK zu einer deutlichen Zunahme des Parkinson-Syndroms, zu Bewusstseinsstörungen und zu autonomen Störungen führen. Bei 80 % der DLK-Patienten treten diese Nebenwirkungen nach Gabe klassischer Neuroleptika auf, bei ca. 54 % sind diese als schwer bis hin zum malignen neuroleptischen Syndrom einzustufen [17]. Vor diesem Hintergrund sind typische Neuroleptika bei DLK kontraindiziert. Ähnliche Nebenwirkungen treten aber auch bei neueren atypischen Antipsychotika auf, sodass auch z. B. Aripiprazol, Amisulprid, Olanzapin und Risperidon nicht empfohlen werden [5]. Falls die Gabe von Neuroleptika trotz aller anderen zur Verfügung stehenden Maßnahmen nicht vermeidbar ist, kommen Clozapin und Quetiapin in Betracht, wobei für beide Substanzen keine größeren klinischen Studien vorliegen. Beide sollen nur in niedriger Dosis gegeben werden (Clozapin: Anfangsdosis: 6,25–25 mg; Erhaltungsdosis: 25–50 mg; Quetiapin: Anfangsdosis: 12,5–25 mg/Tag; Erhaltungsdosis 25–100 mg/Tag). Bei Clozapin sind die notwendigen Blutbildkontrollen und die antcholinergen Effekte zu beachten. Grundsätzlich ist zu beachten, dass der Einsatz der genannten Neuroleptika eine engmaschige Kontrolle und entsprechende Aufklärung der Patienten oder rechtlichen Vertreter über das erhöhte Risiko für Mortalität und insbesondere für zerebro-/kardiovaskuläre Ereignisse erfordert. Die Indikation für den Einsatz von Neuroleptika sollte in regelmäßigen Abständen (ca. 6 Wochen) überprüft werden. Bei fehlender Zielsymptomatik sollten diese Substanzen reduziert bzw. abgesetzt werden.

Kürzlich ist das Antipsychotikum Pimavanserin (selektiver, inverser Agonist am 5‑HT2A-Rezeptor) für die Behandlung der Psychose bei der PK in den USA zugelassen worden. Für den Einsatz bei der DLK liegen nur unzureichende Daten vor [2].

Grundsätzlich muss nicht jedes psychotische Symptom bei DLK behandelt werden. Häufig treten visuelle Halluzinationen ohne wesentliche affektive Begleitreaktion auf. In solchen Fällen kann in einem gemeinsamen Prozess mit dem Betroffenen und den Angehörigen eventuell die Entscheidung getroffenen werden, auf eine Behandlung zu verzichten und die Symptomatik zu tolerieren.

### Hyperaktivität

#### Agitiertes Verhalten/Aggressivität

Unter agitiertem Verhalten wird Unruhe mit erhöhter Anspannung und gesteigerter Psychomotorik verstanden. Häufig tritt auch verstärkte Reizbarkeit mit z. T. konfrontativen Verhaltensweisen, verbaler und körperlicher Aggressivität auf. Agitiertes Verhalten und Aggressivität stellen eine sehr hohe Belastung für Pflegende dar. Oftmals ist eine änderbare Konstellation in der Kommunikation oder der Umwelt auslösend, sodass besonders psychosoziale Interventionen mit genauer Exploration der jeweiligen Bedingungsfaktoren angewendet werden sollten. Eine pharmakologische Behandlung sollte erst in Erwägung gezogen werden, wenn alle nichtpharmakologischen Maßnahmen ausgeschöpft sind [6]. Für die Behandlung der Agitation bei DLK liegen keine randomisierten klinischen Studien vor. Bezüglich des Einsatzes von Neuroleptika gelten die in dem Abschnitt zur Behandlung der Psychose ausgeführten Punkte. Bei der AD liegen Hinweise für die Wirksamkeit von Citalopram bei agitiertem Verhalten vor. Ein Behandlungsversuch kann auch bei DLK gerechtfertigt sein.

### Fallbeispiel

Ein 72-jähriger Patient stellt sich elektiv mit dem Verdacht auf einen Normaldruckhydrozephalus (NPH) in der Klinik vor. Der Patient berichtet von einer zunehmenden „Steifigkeit“. Der Ehefrau fallen v. a. progrediente kognitive Defizite auf. Ihr Ehemann könne sich zunehmend „schlechter konzentrieren“, insbesondere bei Gesprächen mit mehreren Teilnehmern, jedoch sei sein Gedächtnis nach wie vor erstaunlich gut. In einem auswärts angefertigten MRT zeigt sich jedoch nicht das klassische Bild eines NPH (Corpus-callosum-Winkel > 100°, sog. Evans-Index > 0,3), zudem besteht keine temporomesial betonte Atrophie, sondern neben einer zerebralen Mikroangiopathie (Fazekas-Score 2) eine globale Hirninvolution, ohne fokussierte Atrophie. In der klinischen Untersuchung zeigt sich eine sehr leichte, asymmetrische Hypokinesie (links betont). Zudem bestehen eine Hypomimie und ein sehr leichter Rigor des linken Armes bei Koinnervation. Die Okulomotorik ist nicht auffällig, ebenso werden eine Harninkontinenz oder weitere vegetative Symptome verneint. Nur auf Nachfrage wird eine Hyposmie angegeben, zerebelläre Symptome bestehen nicht. Im MoCA-Test erreicht der Patient nur 21 Punkte. In der Liquoranalyse zeigt sich in der Basisdiagnostik ein unauffälliger Befund, jedoch zeigen sich die Degenerationsmarker auffällig (Amyloid42 erniedrigt, Phospho-Tau und Gesamt-Tau erhöht). Es wird die Verdachtsdiagnose einer Alzheimer-Demenz gestellt und eine antidementive Medikation mit einem Acetylcholinesterasehemmer initiiert. Die sehr leichtgradige Bewegungsstörung wird zunächst als vaskuläres Parkinson-Syndrom gewertet.

Bei Wiedervorstellung nach 4 Monaten berichtet die Ehefrau von einer deutlichen Verschlechterung der Kognition. Ihr Ehemann sei an einigen Tagen zeitlich und örtlich nicht mehr orientiert, zudem sehe er Personen aus seinem früheren Berufsleben im Haus, die nicht da seien. Inzwischen wurde ambulant zudem eine Ioflupan(^123^I)SPECT des Gehirns durchgeführt (sog. DATScan), welche ein rechtsbetontes Defizit präsynaptisch lokalisierter Dopaminrezeptoren zeigt. Daraufhin wurde extern eine dopaminerge Medikation initiiert. Aufgrund des unzureichenden Ansprechens der im Verlauf zunehmenden Bewegungsstörungen wurde die Dosis zügig auf aktuell 800 mg/Tag erhöht. Hierunter kam es jedoch zu einer Zunahme der Halluzinationen, sodass die Medikation wieder beendet wurde. Die Frau berichtet, ihr Mann erkenne sie selbst manchmal nicht und wähnt, sie sei eine fremde Person (sog. Capgras-Syndrom). Hierbei sei er sehr aufgebracht. Es gebe jedoch auch Tage, an denen komme ihr der Ehemann recht gesund vor, „wie früher“. Ihr Hausarzt habe zur Beruhigung ein niedrigpotentes Neuroleptikum (Melperon 50 mg) angewendet. Dies führte jedoch wegen einer plötzlichen Bewusstlosigkeit zu einer Vorstellung in der internistischen Notaufnahme. In der erweiterten Anamnese gibt die Ehefrau eine Zunahme des sehr unruhigen Schlafs und der „Albträume“ an, sodass bereits seit 5 Jahren getrennte Schlafräume bestehen. Es wird eine ausführliche neuropsychologische Diagnostik veranlasst, bei der ein mittelgradig demenzielles Syndrom diagnostiziert wird. Es ist v. a. die Visuokonstruktion defizitär, zudem besteht eine markante Störung der Aufmerksamkeitsfunktionen und der psychomotorischen Geschwindigkeit, anterograd-mnestische Defizite sind nicht dominant.

In Zusammenschau der diagnostischen Befunde, der klinischen Symptome und des Verlaufs wird die Diagnose einer DLK gestellt. Es erfolgt zunächst eine Angehörigenberatung durch geschultes Pflegepersonal. Aufgrund der aktuell dominanten inhaltlichen Denkstörungen, der psychomotorischen Unruhe und der wenig ausgeprägten und kaum alltagsrelevanten Bewegungsstörung erfolgt zunächst nur eine Therapie mit Quetiapin 25 mg 1–0–2. Die pausierte Acetylcholinesterasehemmermedikation wird erneut begonnen und konsequent aufdosiert. Hierunter bessern sich die Halluzinationen und die dadurch bedingten Verhaltensstörungen. Im Verlauf erfolgt eine erneute dopaminerge Medikation (keine Agonisten, kein Amantadin, keine COMT-Hemmer), welche die Bewegungsstörung verbessert, ohne wesentlich die intermittierend weiterhin vorkommenden Halluzinationen zu verschlechtern. Die Quetiapinmedikation konnte im Verlauf reduziert werden.

## Fazit für die Praxis

- Bei Patienten mit kognitiver Leistungseinbuße ist es wichtig, die DLK bei den differenzialdiagnostischen Überlegungen zu berücksichtigen.
- Die Behandlung orientiert sich an den Zielsymptomen, wobei neuroleptische Medikamente unbedingt vermieden werden sollten, um schwere Nebenwirkungen zu vermeiden.
- Psychosoziale Interventionen und nichtpharmakologische Behandlungsstrategien sollen immer vor der medikamentösen Behandlung ihre Anwendung finden.
- Cholinesterasehemmer können bei DLK (Off-label-Behandlung) vorteilhaft zur Behandlung von kognitiven Defiziten sowie Störungen bei psychischen und Verhaltenssymptomen eingesetzt werden.
- Im Falle des Auftretens motorischer Symptome kann eine dopaminerge Therapie mit L‑Dopa-Präparaten erfolgen.
